# Construction and validation of a cuproptosis-related five-lncRNA signature for predicting prognosis, immune response and drug sensitivity in breast cancer

**DOI:** 10.1186/s12920-023-01590-z

**Published:** 2023-07-08

**Authors:** Chun Li, Yicong Zhang

**Affiliations:** 1 Department of General Surgery, The First Affiliated Hospital of Anhui Medical University, 218Th Jixi Road, 230022 Hefei, Anhui People’s Republic of China; 2 Hefei Institutes of Physical Science, Chinese Academy of Sciences, Hefei, 230031 PR China; 3 University of Science and Technology of China, Hefei, 230026 PR China

**Keywords:** Breast cancer, Cuproptosis, LncRNA, Prognosis, Drug sensitivity, Immune Response

## Abstract

**Background:**

Despite advances in treatment, recurrence and mortality rates from breast cancer (BrCa) continue to rise, clinical effectiveness is limited, and prognosis remains disappointing, especially for patients with HER2-positive, triple-negative, or advanced breast cancer. Based on cuproptosis-related long noncoding RNAs (CRLs), this study aims to create a predictive signature to assess the prognosis in patients with BrCa.

**Methods:**

The related CRLs RNA-seq data clinicopathological data were collected from The Cancer Genome Atlas (TCGA) database, and the predictive model was constructed after correlation analysis. Subsequently, we examined and validated connections and changes in the CRLs model with prognostic features (including risk curves, ROC curves and nomograms), pathway and functional enrichment, tumor mutation (TMB), tumor immune dysfunction and exclusion (TIDE) and treatment sensitivity.

**Results:**

A prediction model formula composed of 5 CRLs was obtained, and divided breast cancer patients into high and low risk subgroups according to the obtained risk scores. The results showed that the overall survival (OS) of patients in the high-risk group was lower than that in the low-risk group, and the AUC of all samples at 1, 3 and 5 years were 0.704, 0.668 and 0.647, respectively. It was indicated that CRLs prognostic model could independently predict prognostic indicators of BrCa patients. In addition, analysis of gene set enrichment, immune function, TMB, and TIDE showed that these differentially expressed CRLs had a wealth of related pathways and functions, and might be closely related to immune response and immune microenvironment. Additionally, TP53 was found to have the highest mutation frequency in high-risk group (40%), while PIK3CA was found to have the highest mutation frequency in low-risk group (42%), which might become new targets for targeted therapy. Finally, we compared susceptibility to anticancer agents to identify potential treatment options for breast cancer. Lapatinib, Sunitinib, Phenformin, Idelalisib, Ruxolitinib, Cabozantinib were more sensitive to patients in the low-risk group, while Sorafenib, Vinorelbine, Pyrimethamine were more sensitive to patients in high-risk group, namely, these drugs could potentially be used in the future to treat breast cancer patients grouped according to the risk model.

**Conclusion:**

This study identified CRLs associated with breast cancer and provided a tailored tool for predicting prognosis, immune response, and drug sensitivity in patients with BrCa.

**Supplementary Information:**

The online version contains supplementary material available at 10.1186/s12920-023-01590-z.

## Background

Breast cancer (BrCa) is the most common malignant tumor in women. The morbidity and mortality of BrCa are increasing year by year, which seriously disturbs women's health [1, 2]. According to the Global Cancer Statistics report, more than 2,261,419 new cases of breast cancer were diagnosed in 2020, with 684,996 deaths [3]. Although patients with BrCa have received surgery, chemotherapy, radiotherapy, and other treatments, but the prognosis is still not ideal, especially for patients with advanced breast cancer. Therefore, it is of great significance to explore and discover reliable prognostic biomarkers to guide clinical treatment, chemotherapy, immunotherapy, or targeted therapy may be a feasible way to improve the prognosis of BrCa.

Lately, cell death has become a research hotspot, such as apoptosis [4], pyroptosis [5–7], autophagy and ferroptosis [8], which are closely related to tumor recurrence, progression, and metastasis. A recent study has shown that intracellular Cu induces a new form of cell death regulation. As we know, Cu is known to be a trace element in human cells that plays an integral role in maintaining protein function. Excessive Cu in vivo will lead to cytotoxic reactions, but the specific mechanism remains unclear [9, 10]. Accumulating evidence shows that the disruption of copper homeostasis may be related to tumor growth and death, and may promote tumor growth and progression by regulating the tumor immune mircoenvironment [11, 12]. Therefore, cuproptosis is closely related to the occurrence and development of tumors. LncRNA is a heterogeneous transcript with limited affinity for encoding proteins, which affects all kinds of human diseases. LncRNA is an important regulatory factor of tumor diseases, such as mediating tumor cell metastasis, immunogenic response, metabolic regulation and other tumor behaviors [13–17]. Researcheres believe that abnormalities of lncRNAs are closely related to the development and prognosis of breast cancer, and increasingly evidence shows that lncRNAs have the potential to regulate ferroptosis, apoptosis and autophagy in breast cancer [18–21].

Currently, increasing tumor prediction models related to cuproptosis have been found, such as bladder cancer, liver cancer, etc. [11, 22, 23]. However, the application of cuproptosis-related lncRNA combination in prognosis prediction of BrCa patients is still unclear, which deserves our study and attention. Therefore, our study established a CRLs model of breast cancer prognosis that may be related to immune infiltration, providing guidance for the selection of chemotherapy agents in BrCa patients.

## Materials and methods

### Datasets

**Fig. 1:**
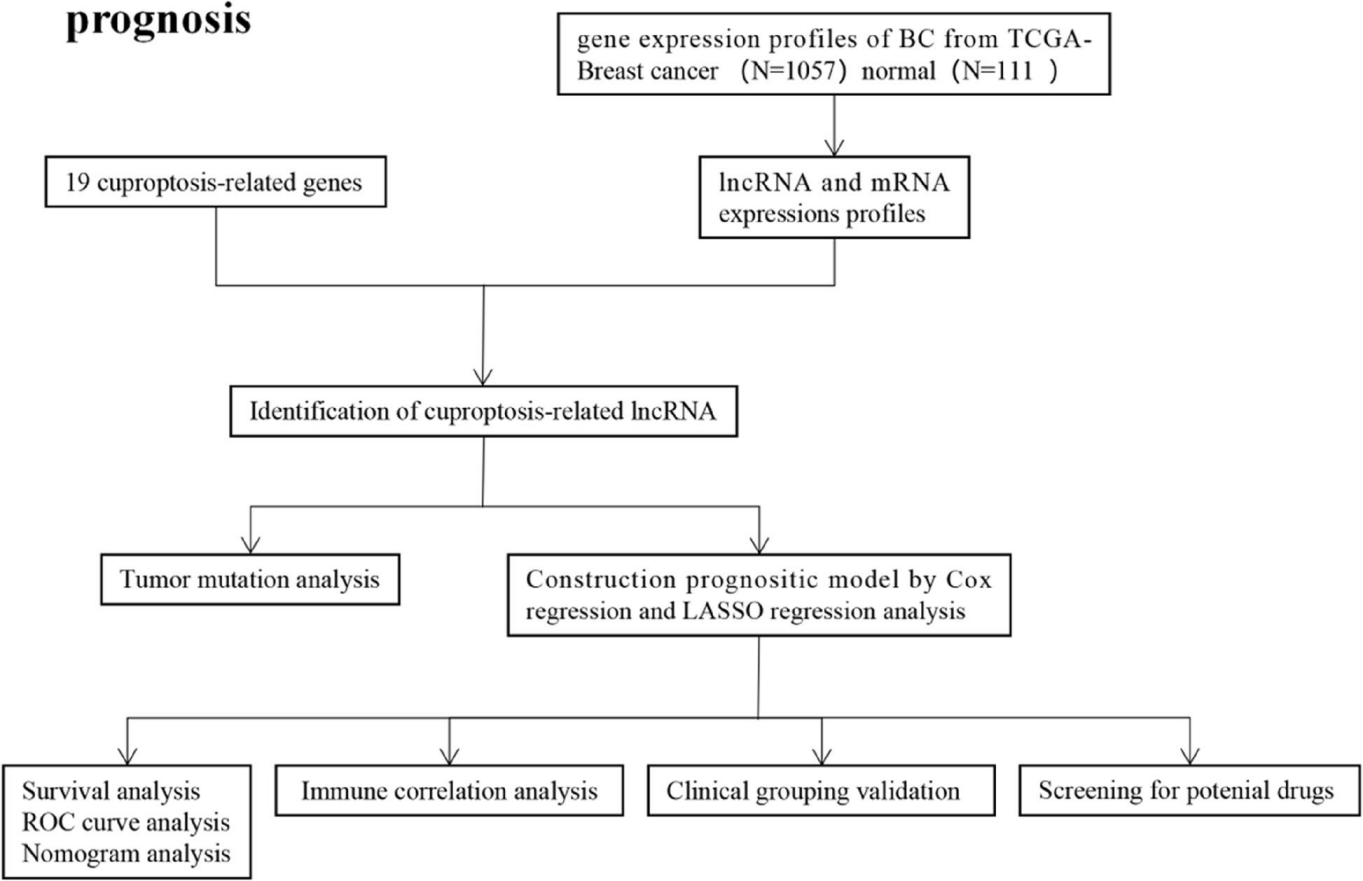
The flowchart of the overall procedure. This flowchart illustrated the process of data collection and analysis for prognostic studies

The design process and grouping were shown as follows in Fig. 1. Downloaded data from the TCGA database (https://portal.gdc.cancer.gov/repository), specimens of 1168 cases, in which the BrCa 1057 cases, normal breast tissue 111 cases, this includes RNA sequencing data of BrCa patient samples, normal samples, clinical information, and tumor mutation Data, and screening for differentially expressed genes between BrCa and normal breast tissue.

### Construction of the CRLs in the TCGA cohort

Nineteen cuproptosis related genes (Table 1) were collected from literatures [9, 10], and related lncRNAs were identified. "Limma" R package was used to detect the correlation between the expression of cuproptosis-related genes and lncRNAs. CorFilter = 0.4 and pvalueFilter = 0.001 were used as screening conditions to obtain the expression of CRLs.

**Table 1:** Cuproptosis-related genes

Gene Name	Gene Name	Gene Name
NFE2L2	ATP7A	LIAS
NLRP3	SLC31A1	LIPT1
ATP7B	FDX1	LIPT2
DLD	PDHB	CDKN2A
DLAT	MTF1	DBT
PDHA1	GLS	GCSH
DLST		

### Construction and validation of the CRLs model of prognosis

Univariate cox regression and least absolute shrinkage and selection operator (LASSO) were used for survival analysis. Screening cuproptosis associated lncRNAs with prognostic value. 964 samples were left in the final cohort for analysis, with 93 clinical samples excluded (including survival time of less than 100 days, and incomplete survival data). The cases were then randomly divided into the train group and the test group. Subsequently, we used the R-package "glmnet" to identify valuable prognostic biomarkers and calculated risk scores for each sample in all datasets based on target gene volume expression. The linear combination of lncRNA prognostic risk score and gene expression level multiplication regression model established the risk scoring formula: $\mathrm{BrCa\,prognosis\,risk\,score }\,(\mathrm{BPRS})= \sum_{(n=1)}^{i}Coefi*Xi$ expression level ($Coefi$ represented the coefficients, $Xi$ indicated the standardized levels of gene expression) (coefi*LncRNA1 exp.) + (coefi*LncRNA2 exp.) + (coefi*LncRNA3 exp.) ….. + (coefi*LncRNAn exp.). In the subsequent survival analysis, the samples were classified into high-risk group and low-risk group using the optimal cutoff value of the risk score analyzed by R language package “survminer”. Subsequently, to explore the prognostic significance of 5-CRLs features in BrCa, Kaplan–Meier analysis was used, and the pheatmap R language package was applied to group by risk score, and risk curves were plotted. Then, to assess whether the risk score model had a good prognosis, univariate and multivariate Cox regressions were used to analyze relevant clinical information. In addition, the predictive power of this risk score was assessed by analyzing characteristics of receiver operator characteristic (ROC) curve using the "timeROC" R language package across all data sets. Finally, this nomogram was established to predict the OS of BCa patients for 1, 3, and 5 years by step-by-step Cox regression model. Besides, PCA components of all genes, cuproptosis-related genes, cuproptosis-related lncRNAs and lncRNA models were mapped, and the scatterplot3D R package analysis method was applied.

### qRT-PCR validation

First, RNA was extracted from human breast tumor tissue and matched normal breast tissue using the TRIzol reagent for analysis. Second, RNA was converted to cDNA using PrimeScript RT Master Mix kit, then qRT-PCR was performed using SYBR qPCR Master Mix. In the end, the **ΔΔ**CT of each sample was calculated and normalized to GAPDH. The sequence of lncRNA primers was shown in Table 2.

**Table 2:** Primer Sequences used in the qRT-PCR assay

Primer Name	Sequence (5’ to 3’)
PRKAR1B-AS1(For)	ATGACATGGGCAGACACAGA
PRKAR1B-AS1(Rev)	AGAGAGGTTCCGAATGCACA
C20rf91(For)	GGGAGGTTTGTGACTAGGCT
C20rf91(Rev)	TCGGAAGACAGCAAGAGTGT
GAPDH(For)	GGAGCGAGATCCCTCCAAAAT
GAPDH(Rev)	GGCTGTTGTCATACTTCTCATGG

### Enrichment analysis and immune correlation analysis

BrCa patients collected in the TCGA cohort were branched into high-risk and low-risk groups according to the median risk score. With log2FC ≥ 1 and p-value < 0.05 as the specific criteria, the differences cuproptosis-related lncRNAs (DECRLs) expression between high-risk group and low-risk group. We then used the "cluster profiler" package to enriched gene pathways, and applied the "GO plot, ggplt2 and circlize" R package to visualize the results. The potential molecular mechanisms or functional pathways were then identified using the immune function gene set files involved in CRLs characteristics through the "GSVA" and "GSEABase" R packages (P-value and q-value/FDR < 0.05 were considered statistically significant).

### Tumor mutation genes and immune escape analysis

First, the tumor mutation burden was calculated by downloading the tumor mutation data from the TCGA database, and the breast cancer patients were divided into high-risk group and low-risk subgroup. Second, the waterfall was mapped using the "maftools" R package (showing only the 15 genes with the highest mutation frequency). Finally, the “survminer” R package was used to calculate the survival curve of the co-morbidity risk for the high-low mutation burden group. We downloaded the tumor escape data from the TIDE website (http://tide.dfci.harvard.edu/), and used "ggpubr" R package for data processing and visualization.

### Screening for potential drugs and susceptibility to breast cancer

To evaluate clinical drug efficacy in BrCa, drug screening was performed using the drug prediction R package 'pRRophetic', and we classified BrCa patients from the TCGA database and calculated the IC50 of commonly used chemotherapeutic agents by using of the algorithm [24, 25] and the corresponding "Predictive" R package. The algorithm allowed users to predict clinical chemotherapy responses by creating statistical models based on gene expression and drug sensitivity data from Cancer Genome Project cell lines using only baseline tumor gene expression data.

### Statistical analysis

R software (v4.1.3) was used for statistical analysis. P-value < 0.05 was considered statistically significant.

## Results

### Construction of differentially expressed CRLs

**Fig. 2:**
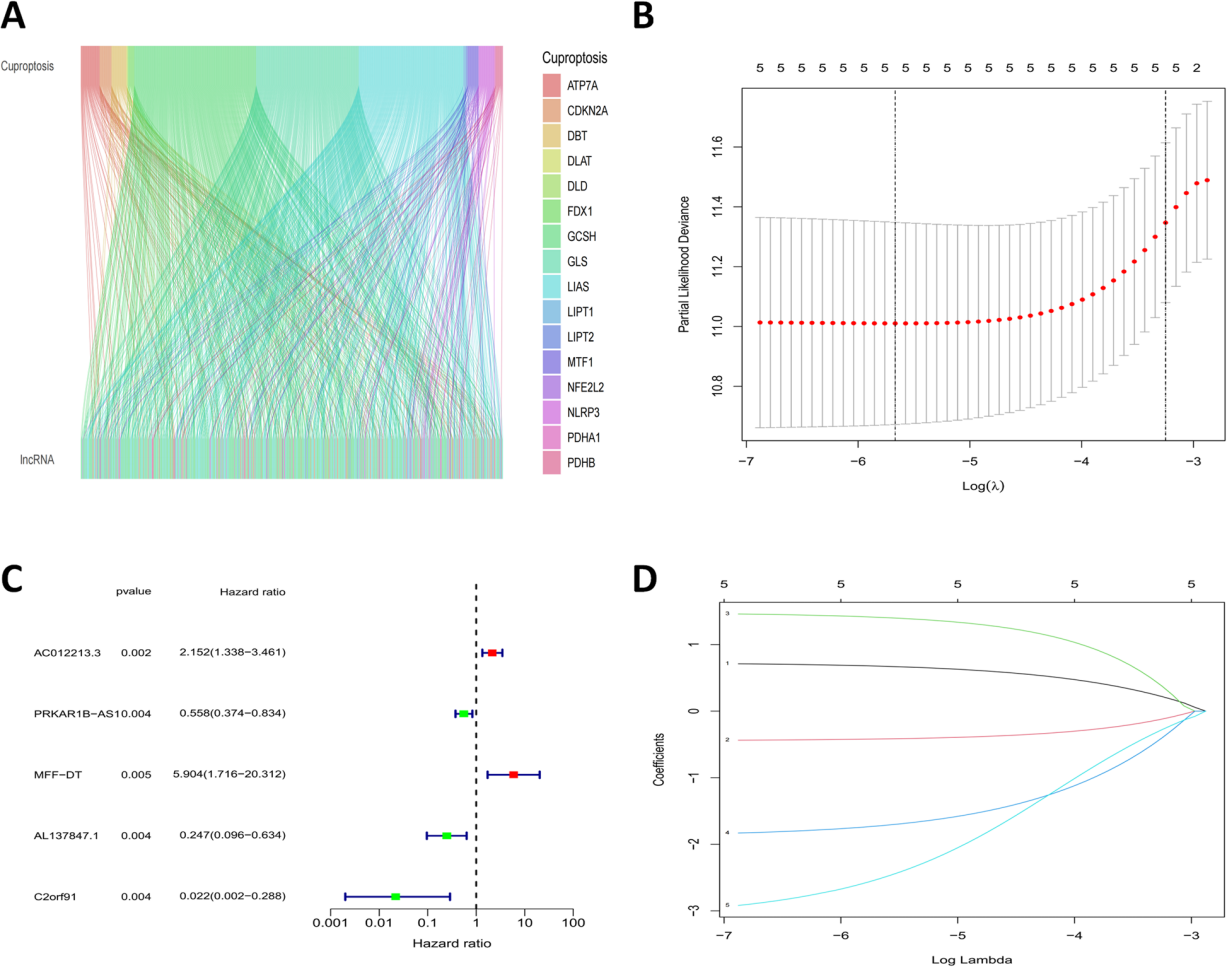
A screen of differentially expressed cuprotosis-related lncRNAs in breast cancer. **A** Correlation analysis results of CRs and lncRNAs Co-expression; **B-D** Univariate cox regression and lasso regression analysis for the prognostic value of CRLs

Firstly, breast tissue samples, were obtained from the TCGA database and then divided into mRNAs and lncRNAs. Subsequently, 19 cuproptosis-related genes (CRs) obtained from the literature were co-expressed with lncRNAs obtained from the database, and the results were compared and correlated and analyzed, and CRLs were finally obtained (Fig. 2A). Additionally, CRLs was associated with the survival time of patients, and patients were randomly divided into the train group and the test group. The clinical characteristics of the two groups were compared later, and no significant difference was found, indicating that grouping was feasible (Table 3). Then, five CRLs (C2orf91(LINC02898), PRKAR1B-AS1, AC012213.3, AL137847.1, MFF-DT) were obtained by univariate cox regression and lasso regression analysis. Then, the model formula was established by using train sets: BrCa prognosis risk score (BPRS) = (-3.11181297991961*C2orf91exp.) + (-0.444042367136264*PRKAR1B-AS1exp.) + (0.725680568783448*AC012213.3exp.) + (-1.88037055291717*AL137847.1exp.) + (1.48270305646497*MFF-DT exp) (Fig. 2B-D).

**Table 3:** Clinical features of breast cancer patients in TCGA database

Feature	Type	Total	Test	Train	*P*value
Age	< = 65	701 (72.72%)	339 (70.33%)	362 (75.1%)	0.1116
Age	> 65	263 (27.28%)	143 (29.67%)	120 (24.9%)	
Gender	FEMALE	964 (100%)	482 (100%)	482 (100%)	1
Stage	Stage I	167 (17.32%)	88 (18.26%)	79 (16.39%)	0.8233
Stage	Stage II	541 (56.12%)	265 (54.98%)	276 (57.26%)	
Stage	Stage III	215 (22.3%)	107 (22.2%)	108 (22.41%)	
Stage	Stage IV	18 (1.87%)	8 (1.66%)	10 (2.07%)	
Stage	unknow	23 (2.39%)	14 (2.9%)	9 (1.87%)	
T	T1	262 (27.18%)	137 (28.42%)	125 (25.93%)	0.3644
T	T2	545 (56.54%)	271 (56.22%)	274 (56.85%)	
T	T3	121 (12.55%)	53 (11%)	68 (14.11%)	
T	T4	33 (3.42%)	19 (3.94%)	14 (2.9%)	
T	unknow	3 (0.31%)	2 (0.41%)	1 (0.21%)	
M	M0	799 (82.88%)	395 (81.95%)	404 (83.82%)	0.5436
M	M1	20 (2.07%)	8 (1.66%)	12 (2.49%)	
M	unknow	145 (15.04%)	79 (16.39%)	66 (13.69%)	
N	N0	442 (45.85%)	233 (48.34%)	209 (43.36%)	0.2108
N	N1	329 (34.13%)	152 (31.54%)	177 (36.72%)	
N	N2	106 (11%)	57 (11.83%)	49 (10.17%)	
N	N3	69 (7.16%)	31 (6.43%)	38 (7.88%)	
N	unknow	18 (1.87%)	9 (1.87%)	9 (1.87%)	

### qRT-PCR validation of five CRLs

**Fig. 3:**
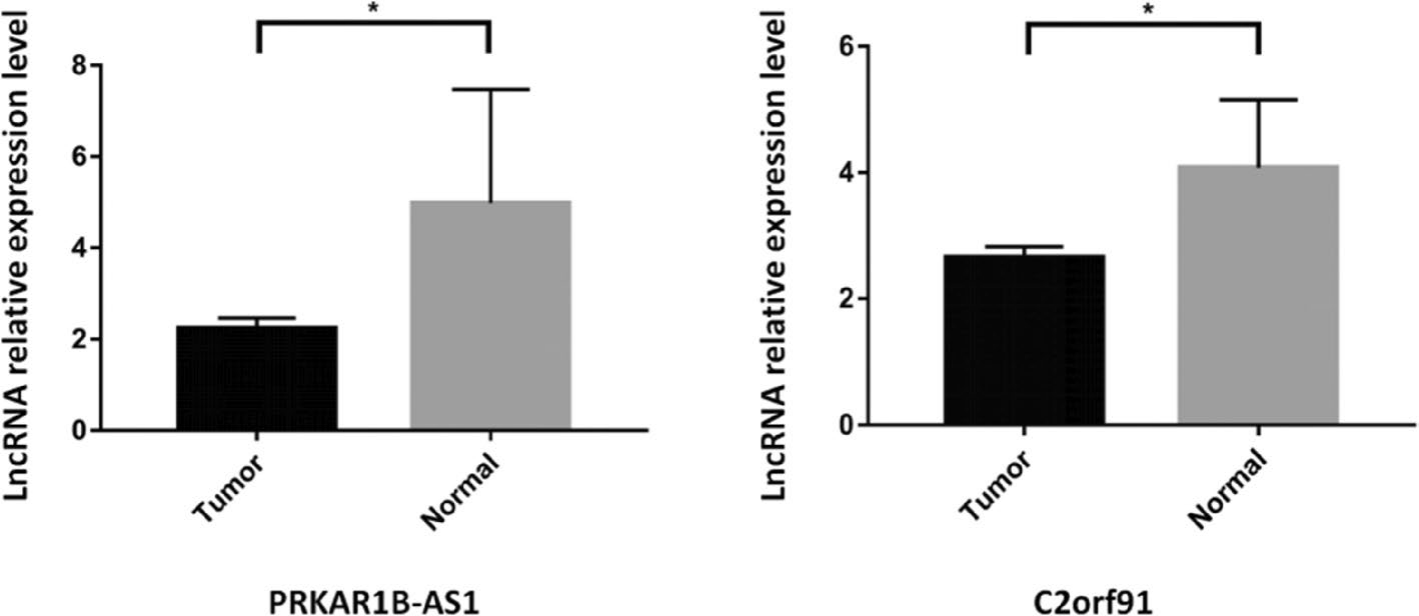
qRT-PCR validation of hub CRLs qRT-PCR validation of CRLs in human breast cancer tissues compared with matched human breast normal tissues.^✳^*P* < 0.05

As AC012213.3 and AL137847.1(2/5 lncRNA), primer serial numbers were not found in literature and database, so they could not be verified for the time being. Therefore, two of the remaining three were selected for experimental verification by qRT-PCR, including PRKAR1B-AS1 and C2orf91 (2/3 lncRNA) in human breast cancer tissues and matched normal breast tissues (Normal breast tissues are assigned to the low-risk group). Results showed that the PRKAR1B-AS1 and C2orf91 lncRNA genes were lowly expressed in human breast cancer tissues compared to matched normal breast tissues. This is consistent with our predictions. The results were shown in Fig. 3.

### The value of prognosis of CRLs signature

**Fig. 4:**
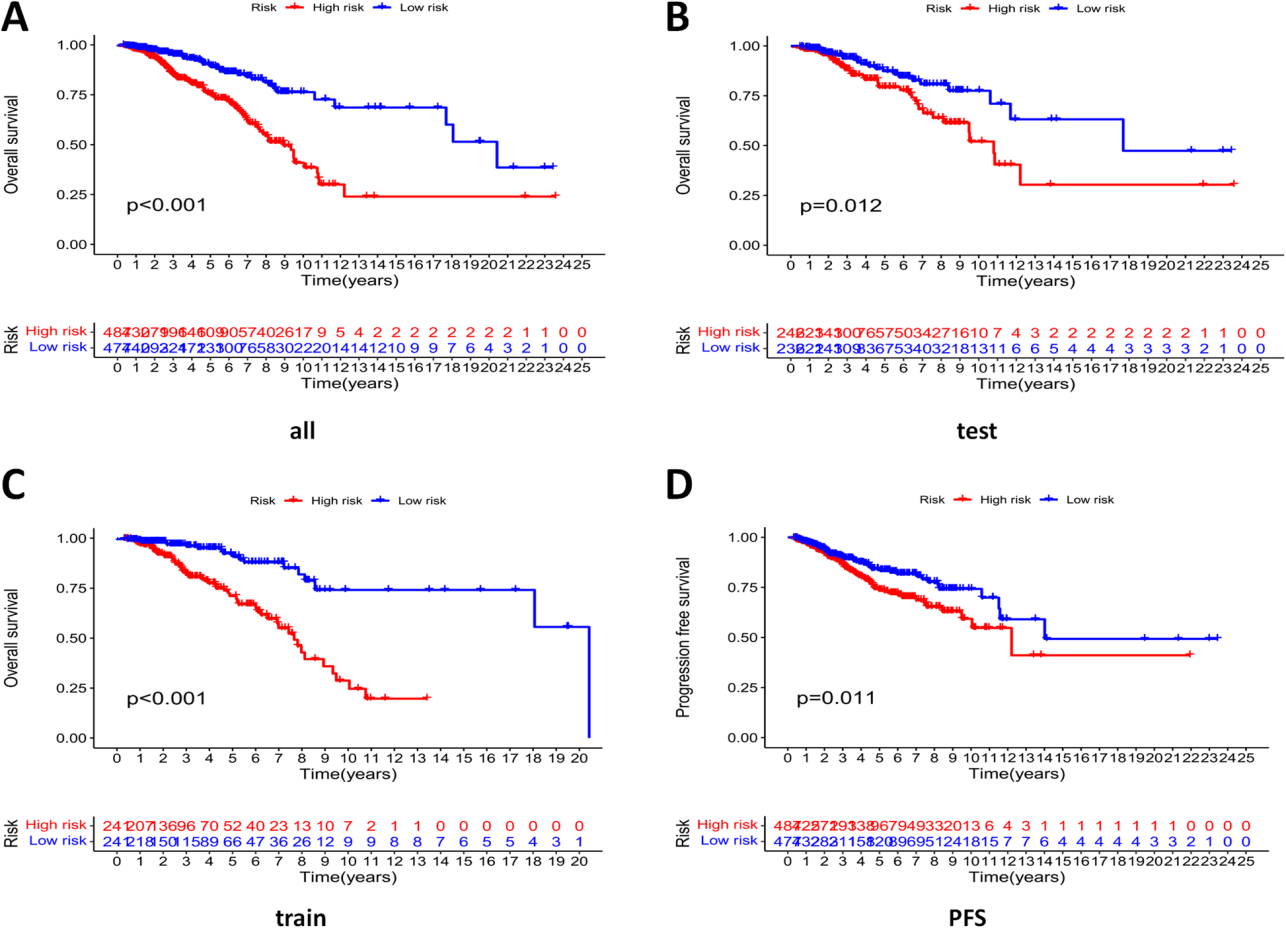
The prognostic value of lncRNA signature. Kaplan–Meier survival analysis compared low risk and high risk groups in all **A**, test **B**, train **C** groups, and PFS **D** in all samples)

According to the obtained risk score calculation formula, the risk scores of all cases in the train group and the test group were calculated, and all cases were randomly divided into low risk group and high risk group. Kaplan–Meier survival analysis showed that the OS and PFS in the low-risk group were significantly longer than those in the high-risk group ( all samples: *P* < 0.001, Fig. 4A; test validation set: *P* = 0.012 < 0.05, Fig. 4B; training set: *P* < 0.001, Fig. 4C; PFS *P* = 0.011 < 0.05, Fig. 4D), indicating the risk signature has a prognostic value.

**Fig. 5:**
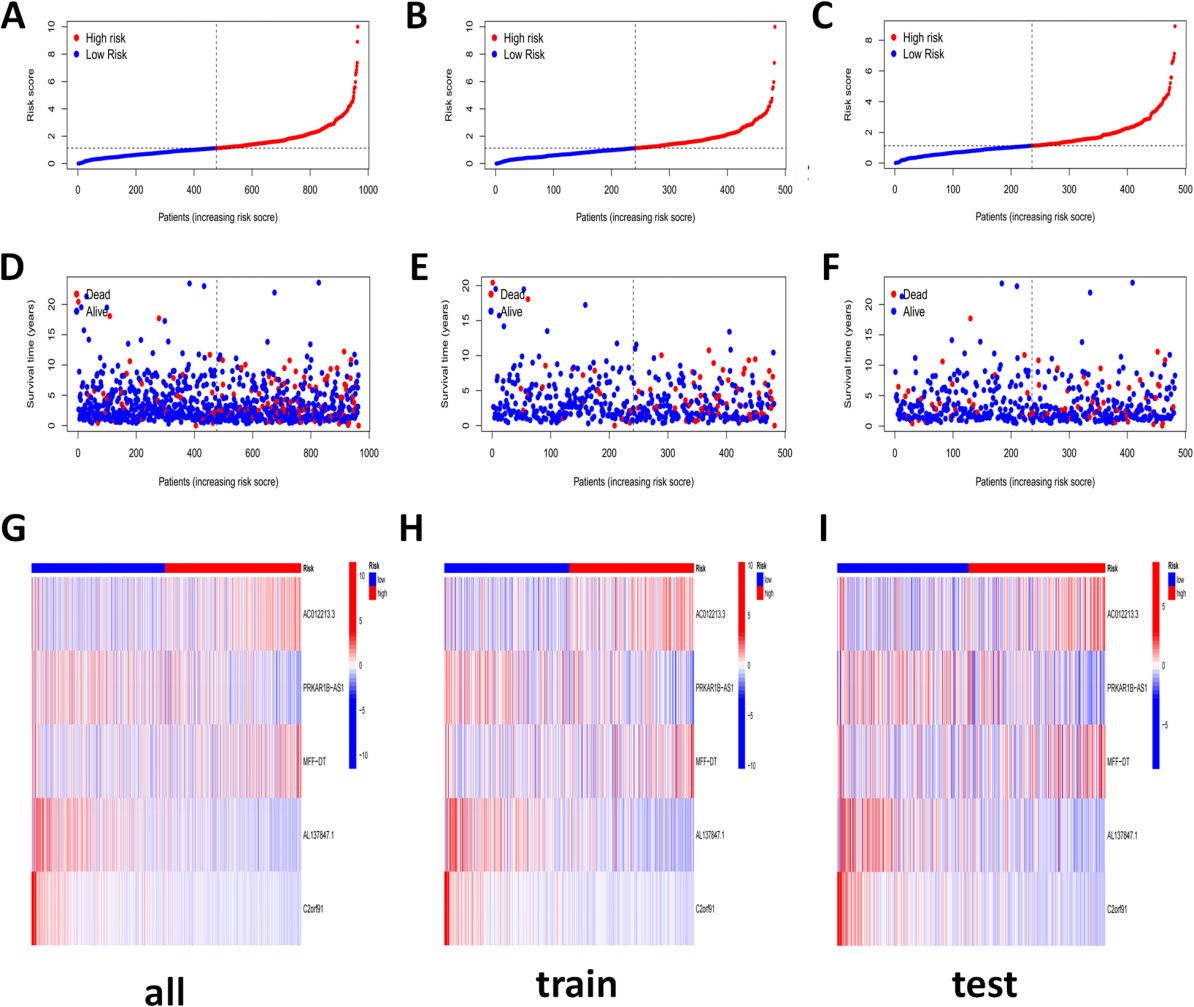
Distribution of BrCa patients based on the risk score. **A**-**F** Risk curve and scatter plot for the risk score and survival status of each BrCa group sample; **G-I** Heat map showing the expression profiles of Cuprotosis-related five lncrnas in all, train and test group samples

Subsequently, a prognostic curve and a scatter plot were used to indicate the risk score and the survival status of all samples, train or test group BrCa patients (Fig. 5A-F). As the risk increased, so did the number of deaths, and most of the deaths were concentrated in high-risk groups (Fig. 5D-F). In addition, the heat map of the expression profiles of candidate CRLs demonstrated that C2orf91, PRKAR1B-AS1, and AL137847.1 were lowly expressed in the high-risk group while AC012213.3 and MFF-DT were highly expressed in the high-risk group (Fig. 5G-I).

### Correlation analysis (prognostic features and clinicopathological features)

**Fig. 6:**
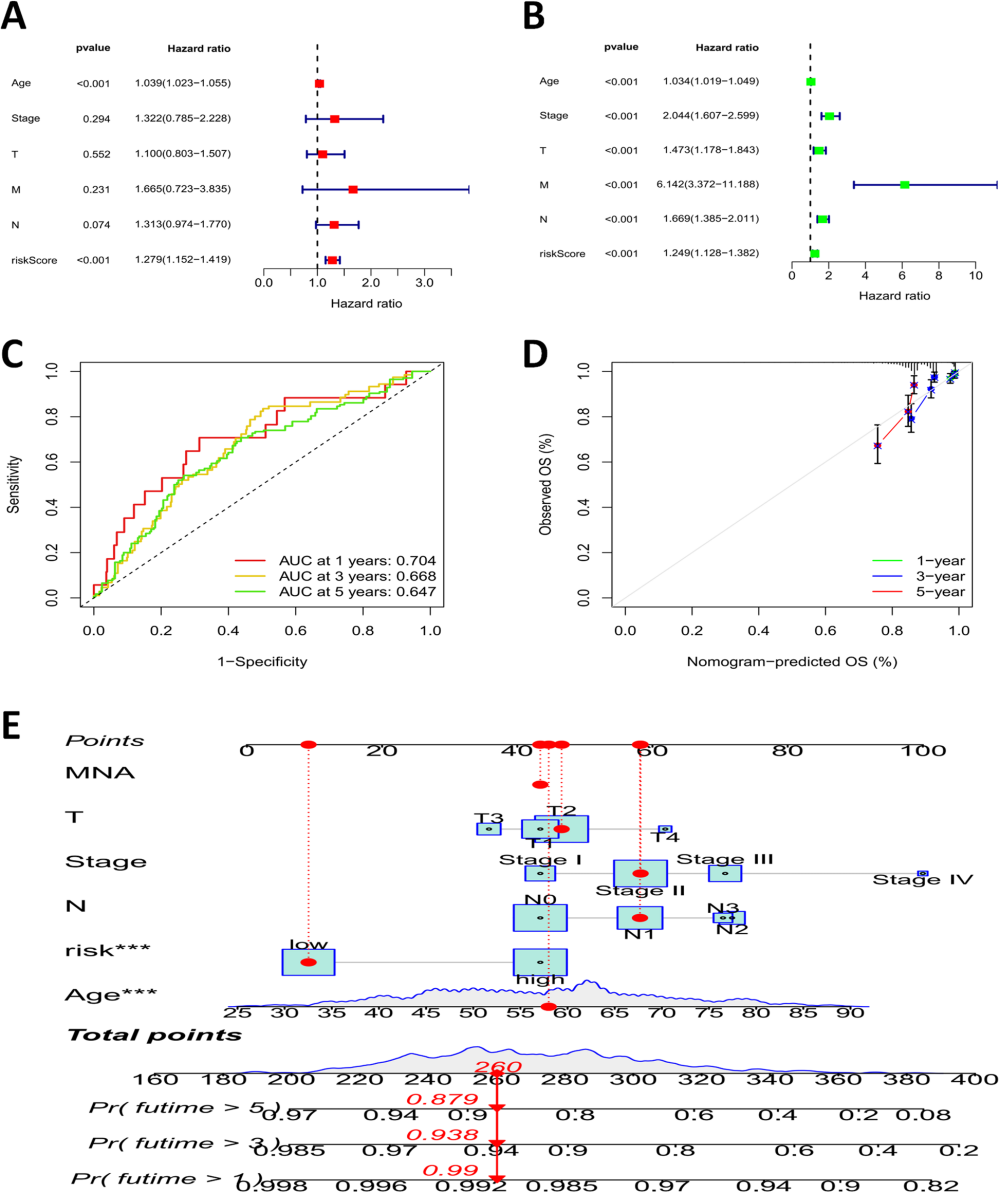
Prognostic value of CRLs signature in TCGA-BrCa cohort. **A**, **B** Univariate and multivariate cox regression analyses for the risk score signature; **C** ROC curves predicted OS of risk characteristics; **D**,**E** A nomogram and its calibration to quantify the 1, 3, and 5-year survival probabilities

Multivariate cox regression was applied to assess the independence of BrCa signatures. The result indicated that the risk model could be used as an unique prognostic factor for predicting OS in BrCa patients, unlike other clinical signatures (Fig. 6A, B). Then, ROC curve was used to evaluate the sensitivity and specificity of risk characteristic model prediction, and the results showed that the AUC of all samples in the first, second and third years were 0.704, 0.668 and 0.647, respectively (Fig. 6C). The areas under ROC curves were all greater than 0.5, indicating high reliability of the results, which further indicated that the prediction model established by us could accurately predict the survival of patients in 1, 3, and 5 years. In addition, a nomogram survival prediction map and its calibration curve was built to calculate survival probabilities at 1, 3, and 5 years applying independent predictors such as risk score, age, and tumor stage (Fig. 6D,E).

**Fig. 7:**
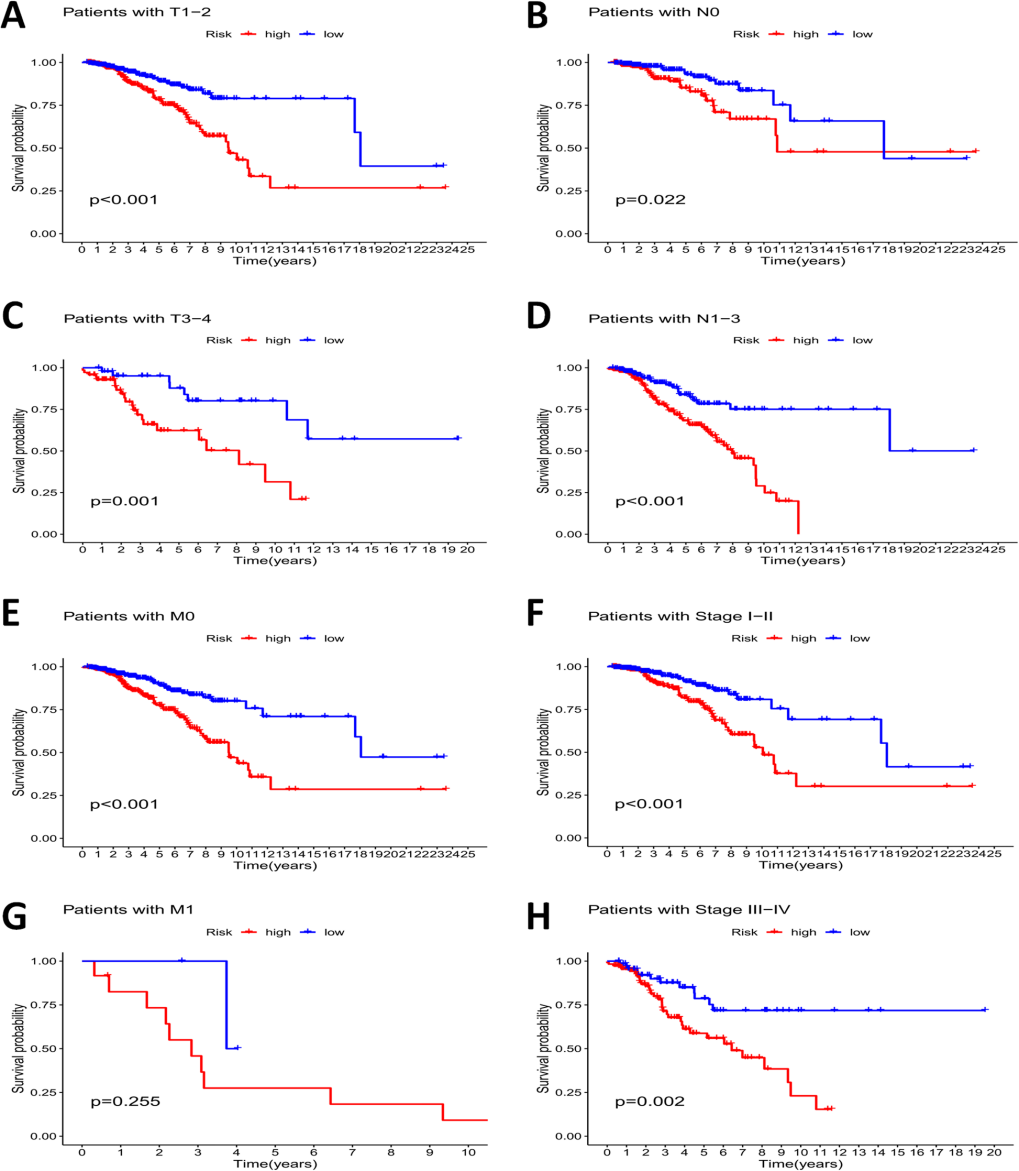
The survival prediction ability of patients with different clinical characteristics of the signature. **A**-**H** Stratified analyses results of tumor stage, T, N, M

Moreover, survival analysis using the following clinical variables, including tumor stage (I-II and III-IV), T(1–2 and 3–4), N(0 and 1–3), and M (M0 and M1) showed that OS was significantly lower in the high-risk group than in the low-risk group, further confirming the critical role of the risk signature in predicting outcome in patients with BrCa (Fig. 7A-H).

**Fig. 8:**
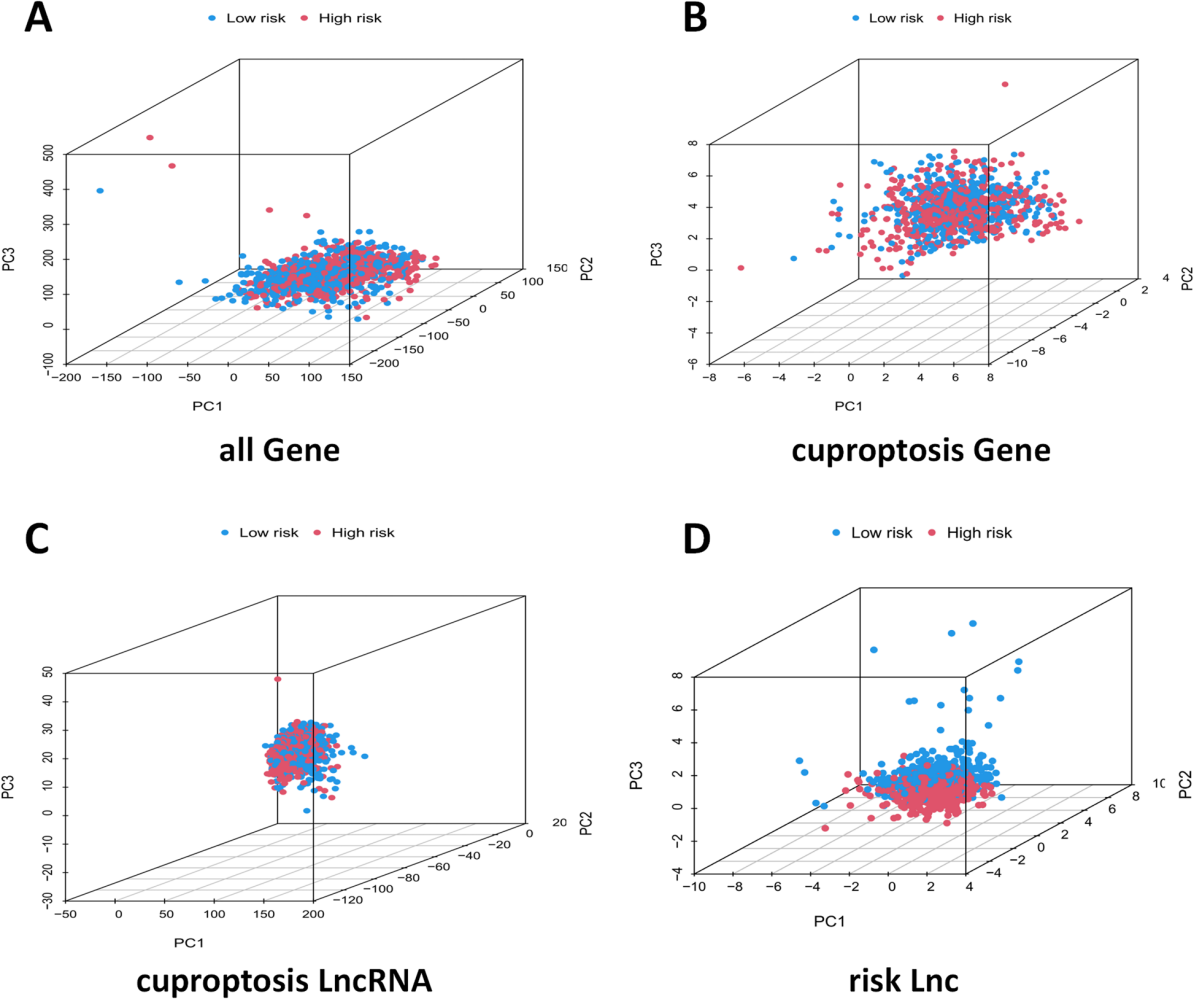
PCA analysis of different model groups in high and low risk patients. **A** all gene group. **B** cuproptosis gene group. **C** cuproptosis lncRNA group. **D** risk lncRNA group

Finally, the result of PCA validated the classification ability of risk CRLs, indicating that the model had an acceptable prediction efficiency and a good degree of distinction and prediction effect between high and low risk groups (Fig. 8A-D).

### Functional enrichment analysis and immune infltration level analysis

**Fig. 9:**
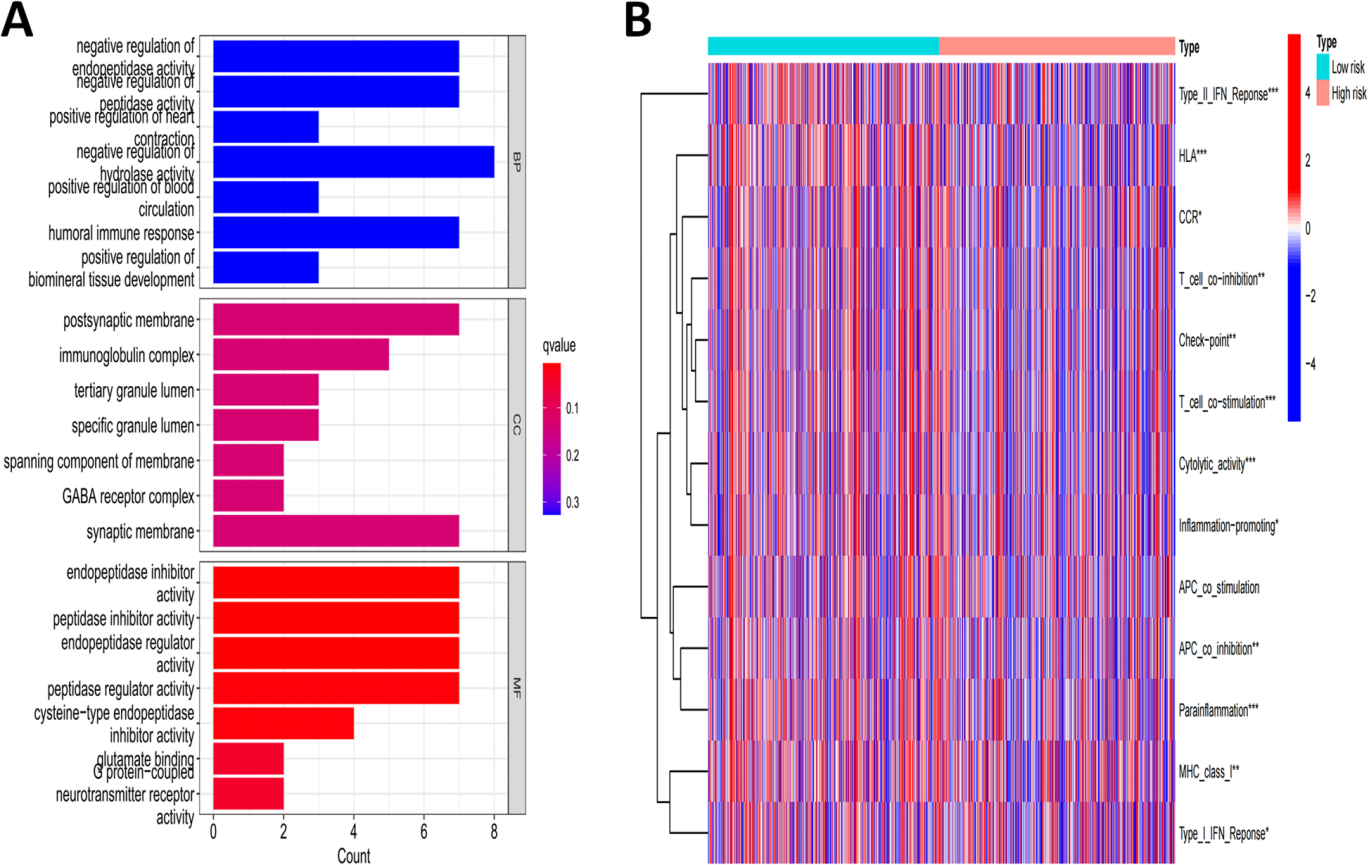
Functional Enrichment Analysis and Immune Infltration Level Analysis. **A** Enrichment analysis results; **B** Heatmap of immune infltration

Set FDR to 0.05 and Log2FC to 1, and obtain the risk difference information according to the expression data file and risk data file (Table S1). Gene set enrichment analysis showed that CRLs were significantly clustered in the regulation of endopeptidase, peptidase, hydrolase activity, humoral immune response, immunoglobulin complex and so on (Fig. 9A). The heatmap of immune infltration based on ssGSEA analysis is shown in Fig. 9B. Comparative analysis of immune cells and pathways confirmed the differences of Type I or II IFN response, check- point, CCR, MHC class I, HLA, T cell co-stimulation, inflammation-promoting, etc..

### Comparison of tumor mutation genes and tumor immune escape in different risk groups

**Fig. 10:**
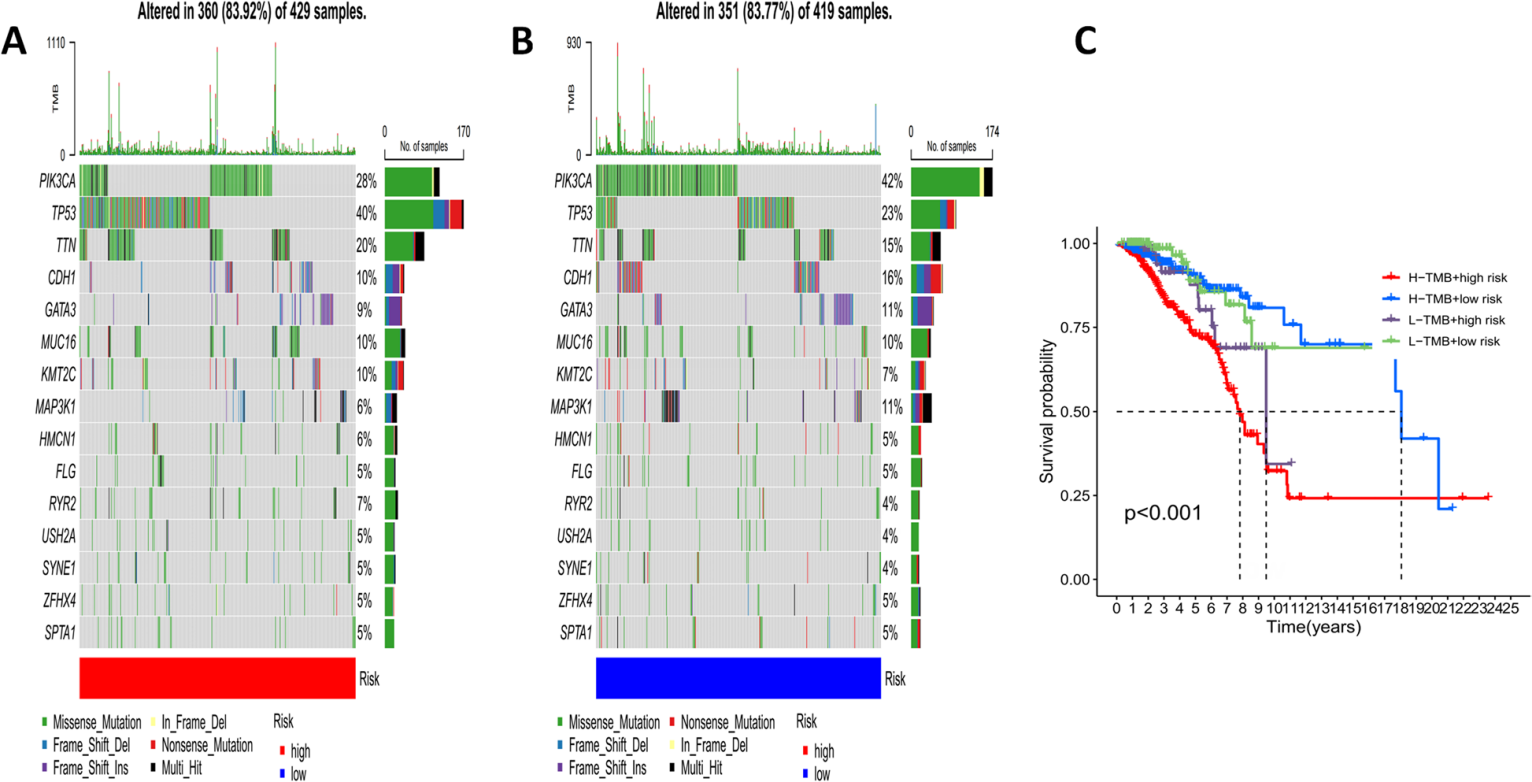
Tumor Mutation Genes and Burden in Diferent Risk Groups Based on Signature. **A**,**B** Detailed mutation information for each gene in the high and low risk group; **C** The survival outcomes combination of patients in the high-low mutation burden group with the high and low risk group

To search the connection between risk score and TMB in BrCa patients (the number of cases meeting the inclusion criteria was 848/964) with cell mutation information. First, the waterfall diagram displayed the mutation information of each gene in detail, with different types of mutation represented by small rectangles in different colors (Fig. 10A,B). From the results, missense mutations in breast cancer tissues were the most common. Then, changes occurred in 360 (83.92%) of 429 high-risk patients, which was similar to 351 (83.77%) of 419 low-risk patients. The mutation frequency of PIK3CA, TP53, TTN, CDH1, and GATA3 ranked in the top 5 of the two groups. TP53 was the highest mutation frequency gene in the high-risk group, and PIK3CA was the highest mutation frequency gene in the low-risk group (Fig. 10A,B). We then used K-M curve to compare the survival outcomes of patients in the high-low mutation burden group. The results showed that patients in the high-risk group, whether they have low or high mutation burden, had the worst prognosis (*P* < 0.001) (Fig. 10C).

**Fig. 11:**
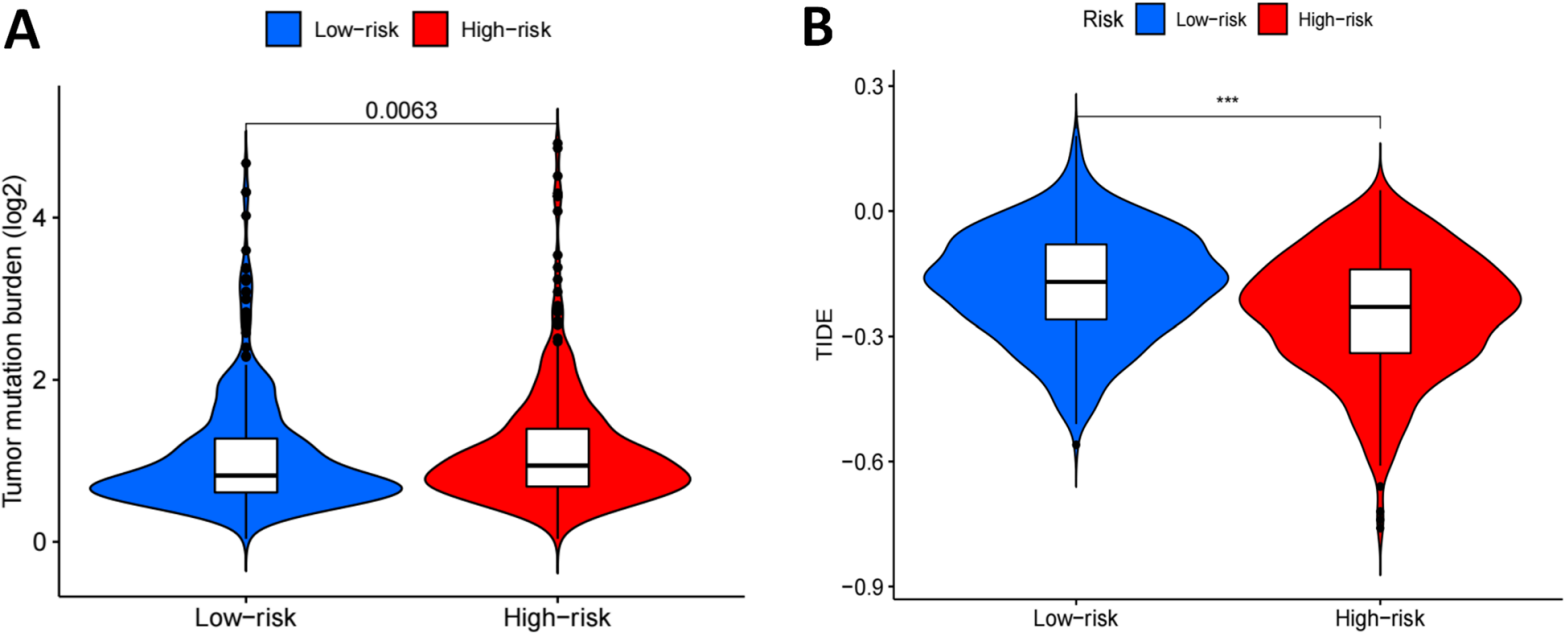
TMB and TIDE in diferent risk groups. **A** The tumor mutation burden outcomes of patients in the high-low mutation group; **B** Comparison of Tumor immune escape scores in two risk groups (***P < 0.001)

Next, the tumor mutation burden (TMB) was lower in the low-risk group than in the high-risk group (*P* = 0.0063 < 0.05) (Fig. 11A). In addition, tumor immune escape information was downloaded from Tumor Immune Dysfunction and Exclusion (TIDE) website (http://tide.dfci.harvard.edu/), and co-analysis showed that tumor immune escape was different between the two risk groups (*P* < 0.001, Fig. 11B), the above results indicated that patients in the high-risk group would yield a better immune therapy response.

### The sensitivity to anticancer drugs between patients in different risk groups

**Fig. 12:**
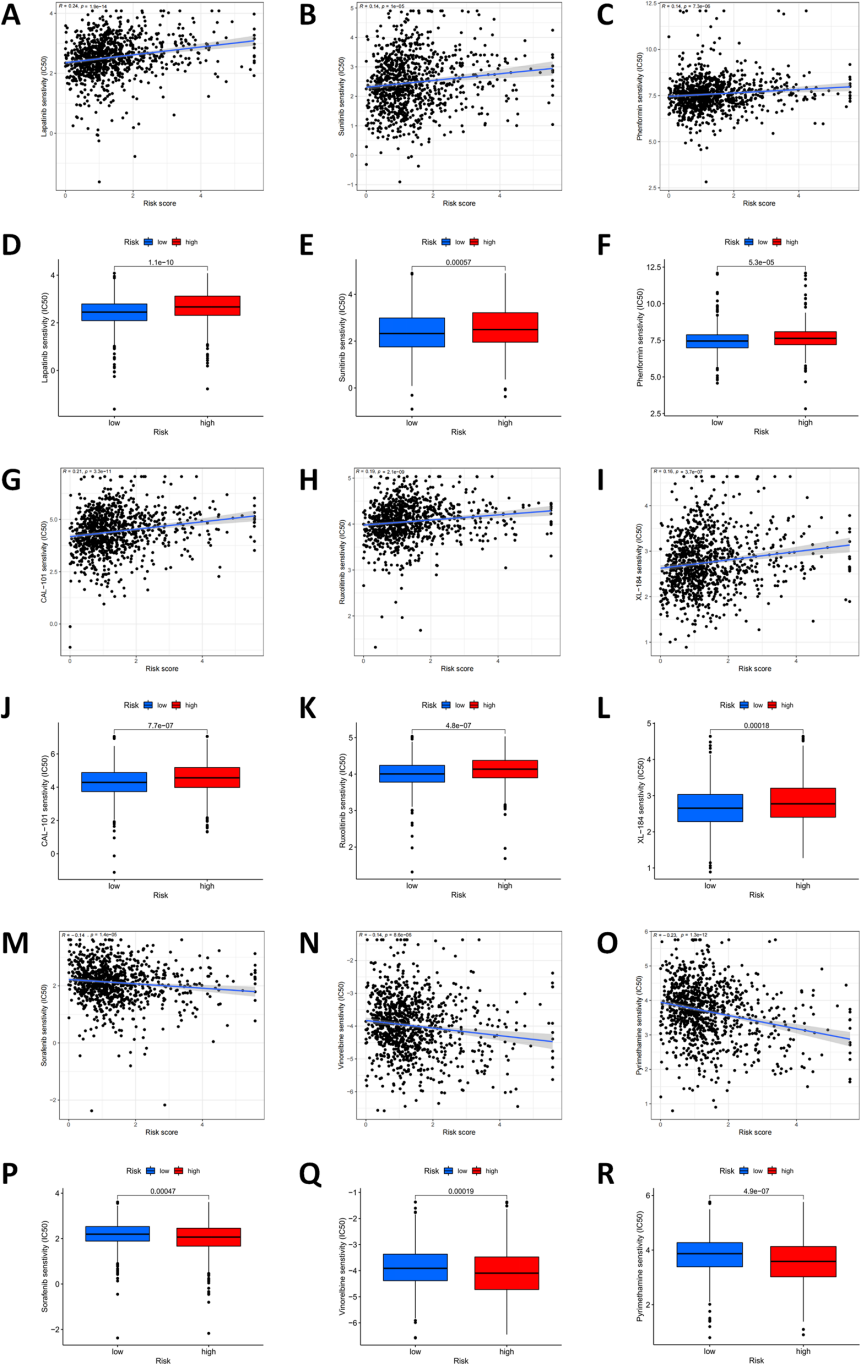
Comparison of the sensitivity of patients with different risk scores to common anticancer drugs. **A**-**R** Results of the IC50 of Lapatinib, Sunitinib, and Phenformin, Cal-101 (Idelalisib), Ruxolitinib, XL-184 (Cabozantinib), Sorafenib, Vinorelbine, Pyrimethamine (*P* < 0.001)

Subsequently, potential treatment options for breast cancer sensitivity to anticancer agents were determined by comparing the high-risk and low-risk subgroups. Results of the IC50 of Lapatinib, Sunitinib, and Phenformin, Cal-101 (Idelalisib), Ruxolitinib, XL-184 (Cabozantinib) were higher in the high-risk group (*P* < 0.001), suggesting that these drugs are more sensitive to low-risk patients, while Sorafenib, Vinorelbine, Pyrimethamine were lower in the high-risk group, indicating that these drugs were more sensitive to high-risk group. In other words, these drugs could potentially be used in the future to treat breast cancer patients with similar conditions (*P* < 0.001, Fig. 12A-R). And this further explains that these drugs had the potential to be used to treat breast cancer patients in the future.

## Discussion

Due to different pathogenic molecules, some patients may have different clinical outcomes despite the same TNM stage or similar risk factors in breast cancer. Therefore, molecular biomarkers for predicting tumor prognosis and drug response are of great significance. Cuproptosis is the latest form of death to be discovered independently of other forms of death. FDX1 and the abundance of lipoacylated proteins, which are key genes for cuproptosis, are highly associated with a variety of human tumors. These results suggestes that copper ion carriers may be a potential therapeutic approach for cancer cells with such metabolic characteristics, which indirectly explains the association with tumor biological behaviors such as proliferation, invasion, and metastasis in cancer, such as breast cancer. Recently, there has been a growing number of studies on the correlation between cuproptosis and tumors, including one on cuproptosis and hepatocellular carcinoma, it is indicated that the key protein FDX1 of copper death is down-regulated in hepatocellular carcinoma, suggesting that it may appear as a protective factor in cancer, and its high expression may be associated with longer survival time. In addition, some scholars have studied the relationship between triple negative breast cancer and bladder cancer and cuproptosis respectively, and the results indicated the potential value of cuproptosis gene in prognosis and treatment [11, 22, 23]. Therefore, cuproptosis-related genes may be related to the occurrence and development of tumor, and may become a new prognostic marker for cancer. Moreover, enormous studies have corroborated that lncRNAs extensively participate in crucial physiological processes such as metabolism and immunity, and are closely related to the occurrence and development of tumors, cardiovascular diseases, nervous system disorders, nephropathy, and other diseases. The application of lncRNAs as biomarkers or intervention targets can provide new insights into the diagnosis and treatment of diseases, they have potential in predicting prognosis, survival, and treatment outcome [26–28], and many studies have shown that lncRNAs play an important role in BrCa [16–19]. Since both cuproptosis and lncRNA are involved in the regulation of tumors, the regulation mechanism of upstream and downstream genes, as well as their relationship with tumor occurrence and development can be better studied by using cuproptosis genes and their co-expression of lncRNAs. Therefore, it is of great significance to identify a cuproptosis-related lncRNA predictive signature in BrCa patients.

In our study, five CRLs were identified for inclusion of the predictive signature. The risk score formula was then used to calculate each patient's risk score, and the BrCa patients were then divided into high-risk and low-risk groups. The OS time of the high-risk group was shorter than that of the low-risk group. The ROC curve showed that the predictive signature had good predictive performance. Besides, the model also provided ideas for exploring related pathways, tumor gene mutations and immune-related functions, drug screening, and treatment. These results indicated that the risk model had a good prognosis prediction and could guide drug selection.

Moreover, there have been increasing studies on CRs and tumors, suggesting that cuproptosis may be involved in the change of tumor microenvironment, which was related to the change of immune function [11, 22, 23, 29, 30], one study linked cuproptosis to bladder cancer identified the role of cuproptosis in the tumor microenvironment by systematically characterizing ten CRs in bladder cancer, and there were differences in infiltration pattern of immune cells, especially T cells and dendritic cells. The predictive score they established could predict immunophenotypic sensitivity to chemotherapy, and immunotherapy response of patients with bladder cancer. In our study, we found that cuproptosis-related lncRNA were also correlated with immune function. To begin with, we performed gene set enrichment analysis of all differentially expressed genes (DEGs) from each patient's genetic risk score (Table S1) in the two risk groups indicated that these DEGs were mainly involved in the regulation of humoral immune response, immunoglobulin complex and so on, which indicated CRLs were strongly associated with immunity.

Additionally, the level of immune infiltration analysis showed that the comparative analysis of immune cells and pathways confirmed that there were significant differences in the high and low risk groups. The results showed that the level of immune microenvironment, including type I or type II IFN response [31], check-point [32], MHC class I [33], HLA, T-cell costimulation [34], inflammation prompting and so on, were differences among the groups and they were statistically significant. Then, we compared the high and low-risk groups of tumor mutation burden and immune escape, and found that the high risk group had a higher tumor mutation burden and a lower immune escape, suggesting that this subgroup might have better immunotherapy effect under the prediction model, however, specific drug selection and therapeutic efficacy of the regimen need to be further clarified in order to better guide clinical treatment. In summary, according to the risk score model, it was indicated that the immune microenvironment of tumor groups on the basis of the risk score of this model might have changed, and the alternation of immune microenvironment would bring changes in immunotherapy strategies, in which the selection of targeted immunotherapy methods and drugs was particularly important.

Besides, we calculated the frequency of mutations, genes, and TMB survival analysis, the result showed that the overall survival rate of the high-risk group was lower than that of the low-risk group, suggesting that the TMB was related to the prognosis of patients. Besides, from Waterfall Figures, we found that the main gene mutations in the two risk groups were the same. However, the mutation order of the first two genes was opposite. The highest mutation frequency of the high-risk group was TP53(40%), and the highest mutation frequency of the low-risk group was PIK3CA(42%) [35–39], it also included an increase in the frequency of other mutated genes, such as TTN,CDH1,GATA3, etc. These mutated genes may be involved in the development of breast cancer, and they are promising to be new targets for BrCa treatment in the future. Finally, we screened chemotherapeutic drugs for breast cancer, especially molecularly targeted drugs. The drug prediction R package 'pRRophetic' was used to assess the drug sensitivity of BrCa therapy to predict therapy response for both risk groups. The IC50 of nine common chemotherapeutic drugs differed significantly between high-risk and low-risk groups, suggesting their drug sensitivities in different groups. Patients in high-risk subgroups may show sensitivity to these agents (Sorafenib, Vinorelbine, Pyrimethamine, *P* < 0.001), patients in low-risk subgroups may show sensitivity to these agents (Lapatinib, Sunitinib, and Phenformin, Cal-101 (Idelalisib), Ruxolitinib, XL-184(Cabozantinib), *P* < 0.001. The current view is that targeted therapy improves disease-free survival and is indispensable for cancer therapy [40], and targeted therapy screening is clearly essential. In this study, several sensitive drugs targeted at high and low risk patients were screened, hoping to bring new benefits to anti-tumor treatment.

The strengths of this study are the systematic analysis based on the TCGA cohort and the evaluation of CRLs in BrCa, and the clinical samples experimental validation of the risk model of human breast cancer tissue, and the results are in line with our expectations. Of course, our study has some limitations, and our prediction model still needs to be validated by a large number of well-designed clinical or in vivo and vitro experiments.

## Conclusion

In summary, this study was conducted a comprehensive and systematic bioinformatics analysis and identified CRLs signatures significantly associated with the prognosis of BrCa patients. This risk score is an independent risk factor for prognosis, immune response prediction, and drug sensitivity screening of BrCa, and have potential application value in the future.

## Supplementary Information

**Additional file 1: Table S1.** Risk differential analysis of all differentially expressed genes in high-risk and low-risk groups.

## Data Availability

Original contributions presented in the study are included in the article/supplementary material. Data were analyzed using TCGA (https: //portal. gdc.cancer.gov). We declare that the data and materials in this study will be provided free of charge to scientists for noncommercial purposes. Further inquiries can be directed to the corresponding author.

## Abbreviations

BrCa: Breast cancer
LncRNAs: Long noncoding RNA genes
CRLs: Cuproptosis-related long noncoding RNAs
TCGA: The Cancer Genome Atlas
OS: Overall survival
CRs: Cuproptosis-related genes
LASSO: Least absolute shrinkage and Selection operator
TMN: Tumor Nodes Metastasis
TMB: Tumor mutation burden
