# Supplementary material for: Construction and validation of a cuproptosis-related five-lncRNA signature for predicting prognosis, immune response and drug sensitivity in breast cancer
Source: BMC Med Genomics. 2023 Jul 8;16:158. doi: 10.1186/s12920-023-01590-z (PMC10329336; doi:10.1186/s12920-023-01590-z)
Supplement: Supplementary file 1 — Additional file 1: Table S1. Risk differential analysis of all differentially expressed genes in high-risk and low-risk groups. [file 12920_2023_1590_MOESM1_ESM.docx]

**Table S1** **Risk differential analysis of all differentially expressed genes in high-risk and low-risk groups**

| gene | lowMean | highMean | logFC | pValue | fdr |
| --- | --- | --- | --- | --- | --- |
| LINC01956 | 3.577939029 | 10.1180822 | 1.499735158 | 2.57E-06 | 1.56E-05 |
| OR4K12P | 3.84233536 | 1.618321971 | -1.247484777 | 6.97E-07 | 5.04E-06 |
| EN1 | 10.23050604 | 27.26632355 | 1.414242679 | 3.86E-05 | 0.000157352 |
| VGLL1 | 12.92346045 | 37.38760986 | 1.53256782 | 2.36E-05 | 0.000103453 |
| LINC02551 | 2.313286233 | 1.069384052 | -1.113163721 | 0.018337151 | 0.032284404 |
| ACAN | 1.491118833 | 7.015370192 | 2.234123999 | 0.005937789 | 0.012186037 |
| CASC16 | 2.518100559 | 0.827026557 | -1.606330335 | 0.002630855 | 0.006018118 |
| TCN1 | 104.7135124 | 37.34623747 | -1.487412813 | 4.70E-06 | 2.62E-05 |
| CALML5 | 202.757019 | 428.5156105 | 1.079595904 | 0.017737164 | 0.031385226 |
| NEK10 | 9.569399092 | 4.140145277 | -1.208746941 | 6.45E-18 | 3.55E-15 |
| FZD9 | 1.166539937 | 3.915734668 | 1.747047311 | 7.00E-06 | 3.68E-05 |
| TPSD1 | 11.82456628 | 5.007999932 | -1.23948082 | 2.13E-06 | 1.33E-05 |
| KRT83 | 0.912792837 | 3.097764887 | 1.762868275 | 0.003578331 | 0.007817545 |
| KRT81 | 97.0784123 | 206.3524619 | 1.087888232 | 0.004198731 | 0.008982915 |
| IGHV3-64D | 79.10073948 | 36.55891123 | -1.113468076 | 0.000923484 | 0.002425778 |
| LINC00589 | 1.391529036 | 0.668458179 | -1.057761803 | 0.000659444 | 0.001812293 |
| AC018816.2 | 1.733231027 | 0.86320024 | -1.005696798 | 9.27E-12 | 3.69E-10 |
| CHST8 | 19.76980342 | 9.42742026 | -1.068363578 | 7.27E-09 | 1.06E-07 |
| TUBA3D | 22.7123297 | 10.61959719 | -1.096746652 | 2.58E-11 | 8.81E-10 |
| ROPN1B | 1.733971174 | 7.017030356 | 2.016780689 | 0.004022625 | 0.008648695 |
| AC073325.1 | 2.228671803 | 0.786516804 | -1.502634686 | 4.61E-09 | 7.19E-08 |
| AL031848.1 | 5.10276485 | 2.519055749 | -1.018396109 | 2.26E-16 | 5.56E-14 |
| TMEM156 | 6.011488889 | 2.294237543 | -1.389707579 | 1.68E-17 | 7.52E-15 |
| TPRG1 | 32.2982116 | 12.90756037 | -1.323237937 | 3.55E-19 | 3.09E-16 |
| FAM3D | 1.858428127 | 4.344369541 | 1.225063933 | 0.010211638 | 0.019499344 |
| DUSP9 | 1.518102725 | 3.301729021 | 1.120952304 | 1.15E-05 | 5.60E-05 |
| EXTL1 | 3.002269322 | 6.691291102 | 1.156231208 | 0.023685006 | 0.040275233 |
| SCARNA6 | 18.73047767 | 3.361178337 | -2.478348696 | 0.000528375 | 0.001496641 |
| RNVU1-15 | 1.925518029 | 0.947461636 | -1.023107199 | 0.002667411 | 0.006087414 |
| SCRG1 | 0.900565723 | 3.188681759 | 1.824056646 | 0.029218007 | 0.04822007 |
| DNER | 1.057907023 | 3.445318686 | 1.703424598 | 2.43E-05 | 0.000105793 |
| CLEC3A | 377.1099663 | 157.9031945 | -1.25594492 | 0.001230011 | 0.003107466 |
| SNORA7B | 4.820342628 | 2.022562047 | -1.252951735 | 0.003425096 | 0.007530563 |
| TRH | 82.93527439 | 31.91380945 | -1.377805017 | 1.88E-06 | 1.20E-05 |
| AC093838.1 | 16.32943686 | 8.106169439 | -1.010382802 | 2.50E-08 | 3.03E-07 |
| RTN1 | 18.16063029 | 9.03542423 | -1.00715003 | 1.81E-17 | 7.77E-15 |
| HNRNPA1P57 | 19.31528613 | 6.69732577 | -1.528085999 | 3.08E-05 | 0.000129027 |
| VMO1 | 26.08249983 | 7.487545483 | -1.800517382 | 2.55E-05 | 0.000110285 |
| CHIT1 | 14.72796649 | 5.742458145 | -1.358817908 | 0.001557633 | 0.003814446 |
| FCER2 | 2.505394025 | 1.064648734 | -1.234660003 | 1.31E-05 | 6.27E-05 |
| IGLV3-12 | 5.737953843 | 2.591472177 | -1.146764459 | 0.011957081 | 0.022344314 |
| PROM1 | 22.50827802 | 46.19172515 | 1.03717874 | 0.000158529 | 0.000531593 |
| SLITRK6 | 93.25560475 | 46.45033768 | -1.005501351 | 0.000217641 | 0.000697675 |
| IGLV1-36 | 51.10931674 | 21.22799428 | -1.267618239 | 0.006387869 | 0.01298555 |
| SLC15A1 | 0.932758316 | 3.841256331 | 2.042003017 | 4.93E-06 | 2.73E-05 |
| AMTN | 2.089227708 | 5.458965572 | 1.385657856 | 0.005655803 | 0.011690074 |
| AD000090.1 | 3.491289553 | 10.35322368 | 1.568248132 | 0.004215498 | 0.009012953 |
| LTF | 1251.326736 | 616.8750478 | -1.020408347 | 1.02E-07 | 9.81E-07 |
| PAX5 | 1.825563382 | 0.8006705 | -1.189061202 | 6.76E-05 | 0.000254456 |
| LINC01768 | 2.1532145 | 0.728586858 | -1.563319168 | 8.54E-15 | 1.11E-12 |
| TUBA3E | 6.471630049 | 2.791154825 | -1.213266985 | 1.69E-09 | 3.07E-08 |
| GALNT5 | 15.73435608 | 6.999200513 | -1.168656094 | 1.11E-06 | 7.58E-06 |
| CA2 | 202.9530813 | 89.16106246 | -1.186660531 | 0.005258431 | 0.010968893 |
| PPP4R4 | 4.265546751 | 1.912108795 | -1.157566063 | 1.20E-06 | 8.08E-06 |
| RNU6-342P | 2.122263138 | 0.771545791 | -1.459779859 | 8.65E-11 | 2.40E-09 |
| ZNF385B | 10.33873036 | 4.55456167 | -1.182674907 | 2.43E-10 | 5.90E-09 |
| PLA2G2D | 10.89420252 | 5.125338569 | -1.087841377 | 0.00224464 | 0.005245005 |
| COL2A1 | 23.26408714 | 54.021186 | 1.215420735 | 0.00062549 | 0.001730492 |
| IGSF1 | 14.37897757 | 5.041525838 | -1.512028753 | 1.49E-05 | 6.97E-05 |
| CCKBR | 0.760367925 | 2.023167214 | 1.411845984 | 0.024595288 | 0.041617564 |
| KLK3 | 1.964557932 | 0.925182854 | -1.086394276 | 3.60E-05 | 0.000148434 |
| TPH1 | 5.350419672 | 2.146656639 | -1.317560608 | 0.000184397 | 0.000603876 |
| SLC1A1 | 66.71453473 | 32.70701513 | -1.028401004 | 3.51E-06 | 2.04E-05 |
| AL591845.1 | 12.92892275 | 6.077645175 | -1.089017718 | 5.73E-13 | 3.55E-11 |
| VIPR2 | 3.448375192 | 1.545084257 | -1.158231238 | 2.60E-12 | 1.25E-10 |
| SHC4 | 2.624083019 | 6.5714718 | 1.324403161 | 0.006915742 | 0.013925019 |
| KCNQ4 | 1.684073201 | 3.423010712 | 1.023310959 | 0.000108176 | 0.000384515 |
| TCL1A | 3.74243812 | 1.745129637 | -1.100644251 | 0.002212419 | 0.005180703 |
| LEMD1 | 2.322528092 | 5.223384463 | 1.169288849 | 0.002669326 | 0.006090943 |
| GRIA2 | 13.902571 | 5.510653354 | -1.335056422 | 8.46E-05 | 0.000310446 |
| AC096589.2 | 2.831035325 | 1.169100513 | -1.27593078 | 1.24E-05 | 5.96E-05 |
| AP001783.1 | 1.025971488 | 3.376244456 | 1.718428726 | 0.000156597 | 0.000526386 |
| LINC02747 | 27.77609801 | 12.57644894 | -1.14311932 | 7.75E-08 | 7.84E-07 |
| GLYATL2 | 34.41277921 | 85.77511622 | 1.317614765 | 2.45E-05 | 0.00010633 |
| UPK1A | 2.412754682 | 6.025018515 | 1.320284438 | 0.030065487 | 0.049411012 |
| IGHV3-72 | 61.11337268 | 24.16822762 | -1.338376425 | 1.48E-05 | 6.94E-05 |
| GAD1 | 1.82356153 | 0.816143395 | -1.159864321 | 0.001014088 | 0.002628674 |
| MT1H | 1.276076205 | 3.452374949 | 1.435874671 | 0.001211097 | 0.003065305 |
| FCRL1 | 1.666312614 | 0.573534565 | -1.538706746 | 9.77E-08 | 9.46E-07 |
| AL596442.3 | 0.892553215 | 1.956841581 | 1.132516874 | 2.51E-05 | 0.000108709 |
| TMC3 | 2.287761775 | 0.597482033 | -1.936969597 | 3.88E-13 | 2.59E-11 |
| AC078882.1 | 8.611699511 | 3.306700616 | -1.380905548 | 0.000167036 | 0.000555833 |
| MS4A1 | 11.66091768 | 5.14356499 | -1.18084079 | 6.77E-06 | 3.57E-05 |
| AC100801.1 | 3.993703459 | 1.532919678 | -1.381445109 | 9.26E-09 | 1.30E-07 |
| TTYH1 | 2.765032635 | 7.320540075 | 1.40465358 | 0.004601542 | 0.009742539 |
| SNORD94 | 14.47552736 | 6.418112697 | -1.173394881 | 0.001188681 | 0.003015498 |
| PICSAR | 1.861980224 | 4.884982444 | 1.391515627 | 0.000964538 | 0.002515226 |
| EPO | 1.206400454 | 3.07316345 | 1.349015623 | 0.00431226 | 0.00919485 |
| BCL2L1-AS1 | 1.490746506 | 0.729204278 | -1.031640025 | 3.77E-06 | 2.17E-05 |
| ACTG1P22 | 7.625614221 | 1.000790828 | -2.929713074 | 0.008531182 | 0.016731281 |
| GSTM5 | 6.732338819 | 2.599248357 | -1.373013296 | 0.003619281 | 0.007896569 |
| AC079296.1 | 1.423266911 | 0.691374709 | -1.041666507 | 1.32E-09 | 2.47E-08 |
| KCNH1-IT1 | 2.441275681 | 1.068082307 | -1.192612395 | 1.43E-10 | 3.76E-09 |
| DHRS9 | 4.732633229 | 1.554215914 | -1.606456181 | 1.45E-06 | 9.57E-06 |
| CST2 | 31.25744322 | 10.0200628 | -1.641308224 | 2.99E-08 | 3.52E-07 |
| TUBAP9 | 2.434224703 | 1.052832923 | -1.20918584 | 0.000169719 | 0.000563853 |
| GABBR2 | 0.666937107 | 1.978575941 | 1.568839815 | 5.23E-05 | 0.000204445 |
| RN7SL314P | 34.74000887 | 14.39180784 | -1.271350294 | 2.80E-09 | 4.71E-08 |
| CD52 | 116.006062 | 49.00755903 | -1.243124001 | 2.65E-07 | 2.23E-06 |
| KIRREL3-AS1 | 1.115559504 | 2.927228508 | 1.391767902 | 1.32E-05 | 6.29E-05 |
| HAO2 | 2.626156045 | 0.409767967 | -2.680073529 | 0.004518826 | 0.009588289 |
| GABRQ | 0.87681464 | 4.544713176 | 2.373845454 | 0.005982506 | 0.012258018 |
| RNVU1-29 | 5.654024843 | 0.866698289 | -2.705676461 | 2.68E-05 | 0.000115091 |
| LINC01238 | 37.05100929 | 13.8846652 | -1.416020454 | 1.80E-08 | 2.28E-07 |
| NKX2-5 | 0.995924983 | 2.284648426 | 1.197863191 | 0.00064978 | 0.001790014 |
| ACTL8 | 4.54798833 | 16.25122649 | 1.837248146 | 8.55E-05 | 0.000313443 |
| AC064799.2 | 2.095341055 | 0.431345072 | -2.28027071 | 5.65E-06 | 3.08E-05 |
| KCNG1 | 1.738701258 | 4.276440554 | 1.298400414 | 6.44E-10 | 1.37E-08 |
| TMEM161BP1 | 1.735546366 | 0.694721971 | -1.320882278 | 2.74E-08 | 3.28E-07 |
| ZFP42 | 1.088954298 | 2.333186277 | 1.099358087 | 0.015991932 | 0.028780949 |
| SNORA11 | 18.75227551 | 8.279579055 | -1.179436345 | 1.99E-05 | 8.93E-05 |
| AC023421.1 | 2.486582739 | 1.164837235 | -1.094036059 | 6.12E-08 | 6.45E-07 |
| SCGB2A1 | 294.6551512 | 113.5856436 | -1.375246988 | 0.009404145 | 0.018176459 |
| SCARNA5 | 70.62916817 | 8.376545722 | -3.075836765 | 0.022539967 | 0.038666351 |
| LRP1B | 3.440979909 | 1.614130561 | -1.092062191 | 1.76E-06 | 1.13E-05 |
| LINC02433 | 1.632838365 | 0.462971389 | -1.818387039 | 0.01088397 | 0.020614053 |
| RN7SL674P | 3.889392872 | 1.712944045 | -1.183066945 | 1.46E-06 | 9.58E-06 |
| SPIB | 6.91874703 | 2.679720979 | -1.368428002 | 0.000130539 | 0.000450721 |
| RHOT1P2 | 1.39118211 | 0.616862971 | -1.173289335 | 2.78E-07 | 2.32E-06 |
| CXADRP3 | 5.799612369 | 2.455404278 | -1.239995896 | 1.36E-05 | 6.48E-05 |
| PCAT18 | 2.222687876 | 0.961359685 | -1.20915716 | 1.54E-16 | 3.91E-14 |
| CABCOCO1 | 3.614402096 | 1.735172519 | -1.058677903 | 1.48E-12 | 7.70E-11 |
| CHRM3 | 0.533117226 | 1.612134702 | 1.59644759 | 0.025054061 | 0.04228564 |
| CHGA | 31.8767261 | 10.62905548 | -1.584490064 | 0.003625625 | 0.007908322 |
| TFPI2 | 37.32983843 | 17.27510551 | -1.111634743 | 1.02E-05 | 5.05E-05 |
| ELF5 | 17.90824343 | 37.53993207 | 1.067802205 | 0.000196719 | 0.000639665 |
| Z97192.4 | 2.710827114 | 0.944832409 | -1.520602749 | 6.84E-05 | 0.000257295 |
| TACC1P1 | 1.483355311 | 0.688387235 | -1.107571961 | 3.85E-05 | 0.000157128 |
| CRISPLD1 | 25.0992624 | 60.81586526 | 1.276802766 | 0.025416371 | 0.04280093 |
| TRIM72 | 2.062481237 | 0.533089049 | -1.951932545 | 6.31E-08 | 6.63E-07 |
| CST4 | 40.01340454 | 17.00841824 | -1.234234407 | 0.000373835 | 0.001113887 |
| AC020907.1 | 1.20458225 | 2.804828576 | 1.219379694 | 0.004086842 | 0.008775346 |
| NKX1-2 | 0.923680887 | 2.276156331 | 1.301133227 | 7.63E-05 | 0.000282857 |
| AL023754.2 | 2.09641188 | 0.554637064 | -1.918306257 | 5.01E-06 | 2.76E-05 |
| CST5 | 103.7459651 | 23.38169182 | -2.149604001 | 5.69E-06 | 3.09E-05 |
| CRABP1 | 28.94290178 | 84.78717426 | 1.550636473 | 1.59E-05 | 7.38E-05 |
| ADCY1 | 21.83413277 | 10.48723987 | -1.057950204 | 4.80E-10 | 1.07E-08 |
| CHAD | 72.6715993 | 33.96514476 | -1.097336653 | 1.91E-14 | 2.26E-12 |
| SLC4A4 | 4.517936827 | 1.687575051 | -1.420712434 | 0.00239179 | 0.005543384 |
| MIR4429 | 1.85122072 | 0.911530938 | -1.022113389 | 7.94E-10 | 1.63E-08 |
| AGTR1 | 47.15933672 | 22.3287153 | -1.078643181 | 1.02E-07 | 9.80E-07 |
| A2ML1 | 4.264385604 | 10.39114764 | 1.284945196 | 2.28E-07 | 1.95E-06 |
| CHRM4 | 1.667697589 | 0.732076694 | -1.187791001 | 1.24E-05 | 5.98E-05 |
| PDZK1 | 46.17210318 | 19.98647656 | -1.20799729 | 2.08E-10 | 5.13E-09 |
| GDF9 | 18.0382385 | 4.530924264 | -1.99318118 | 0.000333006 | 0.001006755 |
| PPP1R14C | 9.960024214 | 20.46743693 | 1.039109295 | 0.000350561 | 0.00105251 |
| AC093297.1 | 85.75591618 | 36.58680633 | -1.228912716 | 1.84E-07 | 1.63E-06 |
| FYB2 | 4.76407935 | 2.279505031 | -1.06347685 | 1.68E-18 | 1.15E-15 |
| GFRA3 | 1.615192662 | 4.404234702 | 1.44718509 | 6.57E-05 | 0.000248388 |
| LCAL1 | 1.190317191 | 3.248538296 | 1.448444644 | 2.89E-05 | 0.000122678 |
| SPAG17 | 2.523121104 | 1.161631485 | -1.119056992 | 4.27E-07 | 3.35E-06 |
| RLN2 | 14.96208522 | 5.211135387 | -1.521641611 | 2.38E-09 | 4.15E-08 |
| IGLV4-3 | 5.296327079 | 1.842333094 | -1.523458295 | 0.0001841 | 0.000603284 |
| TPRXL | 0.86048253 | 1.763141992 | 1.034930858 | 0.000743488 | 0.002010496 |
| PCSK1N | 7.622318239 | 15.55490568 | 1.029067899 | 0.000753679 | 0.002033728 |
| FABP7 | 33.62554591 | 81.25128118 | 1.272832872 | 0.019442736 | 0.03398109 |
